# Regulation of Immune Function by Polyphenols

**DOI:** 10.1155/2018/1264074

**Published:** 2018-04-12

**Authors:** Sujuan Ding, Hongmei Jiang, Jun Fang

**Affiliations:** College of Bioscience and Biotechnology, College of Animal Science and Technology, Hunan Agricultural University, Changsha, Hunan 410128, China

## Abstract

Immune dysfunction is caused by various factors, including changes in relevant immune regulators and environmental stress. Immune system imbalance leads to a variety of diseases in humans. Nutrition may play an essential role in immunity by interfering with proinflammatory cytokine synthesis, immune cell regulation, and gene expression. Polyphenols, one of many categories of natural substances, exhibit a range of biological activities. Polyphenols promote immunity to foreign pathogens via various pathways. Different immune cells express multiple types of polyphenol receptors that recognise and allow cellular uptake of polyphenols, which subsequently activate signalling pathways to initiate immune responses. Furthermore, the polyphenols curcumin and epigallocatechin gallate can induce epigenetic changes in cells. In summary, polyphenols can be used to regulate intestinal mucosal immune responses, allergic diseases, and antitumour immunity.

## 1. Introduction

Immune system function is closely related to human health. Therefore, the pathogeneses of many human diseases involve immune function. This link has led to extensive experimental studies of immune mechanisms in many pathological contexts. Immune dysfunction has many unforeseen consequences. For example, immune dysfunction in the intestinal mucosa triggers diarrhoea in the host and can negatively influence the balance of the intestinal microflora [1]. Accordingly, functional foods, defined as those providing specific nutrition or targeting multiple functional components, are considered a form of preventive medicine [2].

Polyphenols are well-known, pharmacologically active compounds with immunomodulatory activity [3]. This category includes flavonoids, phenolic acids, and stilbenoids, which are ubiquitously produced in plants and exist either as free aglycones or in a state of esterification with glucose and other carbohydrates (glycosides) [4]. Consequently, the absorbed polyphenols interact with the intestinal immune system, leading to both protective and harmful reactions in the host [5]. Polyphenols vary in terms of stability, especially in the context of intestinal digestion. For example, compounds such as anthocyanin and flavonoids are relatively unstable in the duodenum [6], whereas total polyphenols and anthocyanins are generally very stable at simulated in vitro gastrointestinal digestion, with approximate recovery rates of 93% and 99%, respectively [7]. Current evidence strongly suggests that polyphenols contribute to the prevention of several immune diseases. For example, polyphenols in red wine can significantly increase the level of interleukin- (IL-) 21 and decrease the release of IL-1*β* and IL-6 [8]. Furthermore, both a polyphenol-enriched diet and *Ascaris suum* infection were found to modulate porcine mucosal immune responses and gut microbiota compositions [1]. In animal experiments, polyphenols can be administered via the drinking water [9] or gavage into the stomach [10].

In this review, we first introduce the classification and structure of polyphenols and then elucidate the different actions of polyphenols mainly from the perspectives of molecular immunity and epigenetic inheritance. Additionally, we summarise the effects of polyphenols on different types of immune responses.

## 2. The Structure and Function of Polyphenols

**Figure 1:**
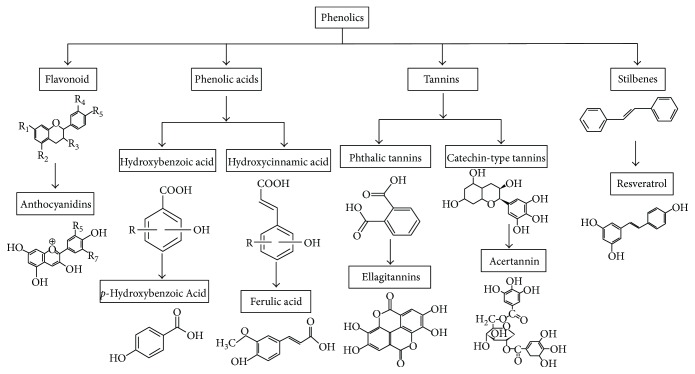
The classification and chemical structures of polyphenols.

Polyphenols are among the most abundant chemicals in the plant kingdom, which yields consumables such as vegetables, fruit, and tea. The polyphenol family comprises a range of molecules with more than 8000 structural variants. These molecules are secondary metabolites of plants and contain many aromatic rings with one or more hydroxyl moieties [11]. Polyphenols are mainly classified by chemical structure and are distinguished from other chemical compounds by the combination of one or more hydroxyl compounds with aromatic rings (phenols). These molecules can be subclassified into flavonoids, phenolic acids, tannins, and stilbenes (Figure 1). In foods, polyphenols are present in complex mixtures and mostly exist as esters, glycosides, or polymers, which are not absorbed in their natural forms.

Dietary polyphenol consumption involves the prohost effect, wherein some (but not all) polyphenols are absorbed in the small intestine. The unabsorbed compounds must be enzymatically hydrolysed by the intestine to facilitate absorption, after which released glycosides with high lipid contents can be taken up by epithelial cells via passive diffusion or active transport.

Flavonoids generally feature a benzophenone structure with two or more aromatic rings, each of which contains one or more phenolic hydroxyl groups connected by a carbon bridge [3]. Phenolic acids are secondary metabolites of plants and fungi and are produced to prevent damage from ultraviolet light, insects, viruses, and bacteria. Additionally, some plant species produce phenolic compounds to inhibit the growth of other plant competitors [12]. Numerous studies *in vivo* and *in vitro* have demonstrated the antioxidant [13], anti-inflammatory [14], and antitumour properties of polyphenols [15]. However, it is important to note that polyphenols differ in terms of the environment in which they are encountered and elicited responses both *in vivo* and *in vitro*.

Phenolic acids can be subclassified as hydroxybenzoic acid and hydroxycinnamic acid, which are, respectively, derived from the phenolic molecules benzene and cinnamic acid [16]. Phenolic acids are organic carboxylic acids that each contains a phenolic ring, which is equipped with the C6-C1 of the *p*-hydroxybenzoic acid or the C6-C3 of the hydroxycinnamic acid. The maximum absorption peaks of *p*-hydroxybenzoic acid or hydroxycinnamic acid are detected at 280 and 320 nm, respectively [17, 18]. Ferulic acid inhibits the production of tumour necrosis factor- (TNF-) *α* in RAW264.7 cells stimulated with lipopolysaccharide (LPS) [19, 20]. Immunological studies have shown that phenyl ethyl caffeate strongly and significantly inhibits the expression of interferon gamma-induced protein- (IP-) 10 in response to TNF, as well as the production of lymphoid factors and activation of nuclear factor- (NF-) *κ*B [21–23].

Stilbenes are a class of compounds characterised by a 1,2-diphenylethylene skeleton. These compounds exhibit extraordinary potential in the biomedical field. For example, the stilbene resveratrol is potentially very beneficial for human immunity and antioxidative mechanisms. Resveratrol has been shown to directly target central cellular components of innate and adaptive immunity, such as macrophages, large lymphocytes, and dendritic cells (DCs). Furthermore, previous research has identified few significant adverse effects of resveratrol [24, 25]. In animal experiments, resveratrol exerts an immunomodulatory effect by decreasing the expression of the activating receptors CD28 and CD80 on immune cells and increasing the production of the immunosuppressive cytokine IL-10. Tannins were originally identified in astringent plant extracts, prior to chemical structure analysis. Tannins have since been divided into two subgroups according to the type of polyphenol group within the molecule: phthalic tannins and catechin-type tannins [26].

## 3. Polyphenols Use Various Immunomodulatory Mechanisms

Decades of research on polyphenols have led to several insights regarding the effects of polyphenols on immune function. Each type of polyphenol targets and binds to one or more receptors on immune cells and thus triggers intracellular signalling pathways that ultimately regulate the host immune response. Dietary interventions that involve polyphenols may modulate immune responses by affecting epigenetic mechanisms, such as regulatory DNA methylation, histone modification, and microRNA-mediated posttranscriptional repression that alter the expression of genes encoding key immune factors.

Immune cells express many receptors that allow the transmission of external stimuli to activation processes within the cell *in vivo*. Currently, researchers are studying a range of polyphenol receptors. Epigallocatechin gallate (EGCG) targets three different cellular receptors: the 67 kDa laminin receptor (67LR), zeta chain-associated 70 kDa protein (ZAP-70), and retinoic acid-inducible gene (RIG-I) [27, 28]. Of these, 67LR is expressed by neutrophils, monocytes/macrophages [29, 30], mast cells, and T cells [31, 32] and regulates the adhesion and inflammatory processes of these cells. EGCC has the ability to inhibit the activity of ZAP-70 by inhibiting the T cell-induced pathway mediated by CD3 in the leukemic cells [33]. The signal transduction pathway downstream of RIG-I triggers the interferon reaction [34]. The aromatics receptor (AhR), which is also known as the naringin and dioxin receptor, is a member of the alkaline helix-ring-helix/Per-Arnt-Sim homologous family and the receptor of naringenin. Dietary flavonoid naringin induces regulatory T cells through AhR-mediated pathways [35]. These receptors appear to be involved in various types of toxicity [36, 37]. The transcription factor specific protein 1 (Sp1) is strongly expressed on many cancer cells [38]. Resveratrol effectively inhibits tumor growth by inhibiting Sp1 expression and inducing apoptotic cell death, and Sp1 becomes a novel molecular target for resveratrol in human malignant pleural mesothelioma [39]. The Toll-like receptor (TLR) 4, T cell receptor (TCR) *αβ*, and IgM- (sIgM-) B-cell receptor are receptors for baicalin (BA) on T and B cells, and BA can regulate innate and adaptive immune regulation by upregulating those immune receptors [40]. These receptors change under specific conditions to regulate immune factors in the host [40].

Many studies have investigated the effects of polyphenols on various types of immune cells, such as primary macrophages, to identify potential targets [41, 42]. One research group used healthy peripheral blood mononuclear cells (PBMC) as a model in which to monitor NO production. Their results demonstrated that red wine could induce NO production by human monocytes and that the vasodilatory actions of the subsequently released NO could prevent atherosclerosis [43]. Moreover, dihydroxyl phenolic acid, a product of microbial metabolism, exhibits anti-inflammatory properties *in vitro*; specifically, it reduces the secretion of TNF-*α*, IL-1*β*, and IL-6 from the PBMCs of healthy subjects [44]. DCs are the most effective antigen-presenting cells (APCs) in the innate immune system. These cells act as key immune sentinels with the unique ability to integrate and deliver large quantities of incoming signals to lymphocytes and thereby initiate and regulate an adaptive immune response [45]. Some studies have found that polyphenols affect various aspects of DC biology, such as differentiation [46] and maturation [47], and the underlying mechanisms have been partially elucidated. TLR connections induce the activation of the mitogen-activated protein kinase (MAPK), Akt, and nuclear factor- (NF-) *κ*B pathways, leading to DC activation [47, 48]. Regulatory T cells (Tregs) contribute to the maintenance of immune tolerance and, therefore, the inhibition of autoimmunity [49].

As noted, dietary components can selectively activate or inactivate gene expression via epigenetics, wherein gene expression is altered without changing the underlying DNA sequence [50]. Diet and other environmental factors can cause epigenetic changes with potentially important immune effects [51]. The findings of many studies emphasising the importance of the environment in terms of epigenomics support the concept that maternal influence via dietary habits may cause permanent epigenomic changes in the offspring [52, 53]. Polyphenols can modulate epigenetic patterns by altering the levels of *S*-adenosylmethionine and *S*-adenine isoforms or by directing factors involved in DNA methylation and histone modification [54]. Curcumin (diferuloylmethane), a component of turmeric (*Curcuma longa*), has recently been identified as an inducer of epigenetic change [55]. EGCG can affect the epigenome by inhibiting DNA methyltransferase-1 (DNMT1) and gene transcription [56]. DNA methyltransferases (DNMTs) comprise a family of enzymes that methylate DNA at the C5 sites of cytosine residues, and the inhibition of these enzymes has been shown to effectively treat various developmental and proliferative diseases [57]. The main type of polyphenol in green tea, EGCG, inhibits DNMT activity and reactivates gene methylation (i.e., silencing) in cancer cells [58]. Furthermore, epigenetic regulation mediated by polyphenols also affects microRNA expression in various biological processes in multiple cell types [59, 60]. A dietary intervention study conducted in apoE-deficient mice demonstrated that nutrient doses of polyphenols could regulate microRNA expression in the liver. Analyses of microRNA targets and mRNA pathways suggest that polyphenols can regulate cell functions at both levels [61].

## 4. Regulatory Effects of Polyphenols on Different Immune Responses

Polyphenols vary in terms of source and type (e.g., tea polyphenols, red wine polyphenols, the polyphenolic fraction of *Cinnamomum zeylanicum* bark (PP-CZ), and E polyphenols), as well as functions. The following section mainly discusses the effects of polyphenols on intestinal mucosal immunity, allergic reactions, and antitumour responses.

### 4.1. Polyphenols Regulate the Intestinal Mucosal Immune Response

Polyphenols are bioactive substances that promote intestinal health via various mechanisms, such as the regulation of mucosal immunity and inflammation. The gut innate immune system contains three lines of defence: the mucosal layer, epithelium, and lamina propria. The mucosal layer is the first line of host intestinal defence against foreign pathogens [62]. Many studies of the regulatory effects of polyphenols on intestinal immune function have yielded powerful evidence and warranted subsequent studies. The nutritional protection of polyphenol-induced abnormal crypt lesions may represent a key step in the prevention of gastrointestinal tract tumours via decreasing abnormal crypts [63]. Polyphenols derived from plums may target the Akt/mTOR pathway and microRNA 143, both of which have been identified as potential factors in colon cancer tumorigenesis [64]. Gastrointestinal helminths are among the most common pathogens affecting both humans and livestock worldwide. *In vivo* experiments have shown that polyphenols enhance intestinal mucosal immunity by increasing the populations of intraepithelial T cells and mucosal eosinophils, as well as the propionate concentration in the distal colons of pigs infected with *Ascaris suum* [1]. Curcumin significantly increased the immune index of IgA in the guts of rats fed a high-fat diet [65]. Cocoa has been shown to modulate gut immune responses in young mice by increasing the percentage of *γδ* TCR T cells and lowering the effect of IgA [66, 67].

### 4.2. Polyphenols Regulate Allergic Diseases

Allergic disease affects humans at all stages of life (i.e., new-born to elderly) and often has a genetic predisposition. Many factors appear to contribute to the development of allergies [68]. Polyphenols have been identified as immune regulators with anti-inflammatory effects [69]. Quercetin, which is expressed widely in plants, is a flavonoid compound with multiple pharmacological effects [70]. Polyphenols, which possess the well-known ability to scavenge free radicals, also exhibits antiallergic effects, including inhibition of histamine release, reduction of proinflammatory cytokines, and leukocyte production [71]. Polyphenols have also been shown to regulate the Th1/Th2 balance and inhibit antigen-specific IgE antibody formation. Two mechanisms may be involved in this process. First, polyphenols may affect the formation of the allergen-IgE complex [72]; second, these compounds may affect the binding of this complex to its receptors (FceRI) on mast cells and basophils [73]. The ingestion of tannins isolated from apples has been shown to prevent the development of food allergies, and this effect may be associated with an increase in the proportion of *γδ* TCR T cells in intestinal intraepithelial lymphocytes [74].

### 4.3. Antitumour Effects of Polyphenols

Although molecular targets of polyphenolic anticancer activity have been detected, strong evidence also suggests that polyphenols may also exert anticancer activities through immune-mediated mechanisms. Immunogenic cell death (ICD) is defined as a pattern of cell death that stimulates an immune response to an antigen from a dead cell, particularly a cancer cell [75]. The strength of a response to ICD is mainly mediated by damage-associated molecular pattern molecules, which include the surface-expressed molecule calreticulin and the secreted molecules ATP and high-mobility group protein B1 (HMGB1) [76]. Gomez-Cadena et al. [77] found that a gallotannin-rich fraction obtained from *Caesalpinia spinosa* (P2Et) induced spontaneous tumour cell apoptosis, as determined by the activation of caspases 3 and 9, mobilisation of cytochrome C, and externalisation of annexin V on the cell surface. In a subsequent experiment with C57BL/6 mice, the protective effects of P2Et treatment were abolished in immunodeficient mice and were reduced following the depletion of CD4^+^ and CD8^+^ T cells. These results suggest that the antitumour activity of P2Et requires the immune system and is at least partly T cell-dependent. Moreover, many studies have proven the antitumour activities of polyphenols. Polyphenol E inhibits the tumour growth by targeting both myeloid-derived suppressor cells (MDSC) and CD8^+^ T cells. *In vitro*, polyphenol hinders MDSC development and migration, promotes the differentiation of these cells into a more neutral form via signalling through 67LR, and induces the expression of the granulocyte colony-stimulating factor [77]. Dietary polyphenols induce cancer cell apoptosis by enhancing signalling through the TNF-related apoptosis-inducing ligand- (TRAIL-) mediated apoptotic pathway [78].

## 5. Conclusion

With the discovery and utilization of functional foods, studies have increasingly investigated more suitable candidates among natural products. Polyphenols have been shown to enhance antitumour immune activity, as well as immunomodulatory processes and intestinal mucosal immunity. Many studies have explored and verified the biological activities of polyphenols *in vivo* and *in vitro* and have consequently elucidated many of the underlying regulatory mechanisms. Regarding the future development of polyphenols as immune factors, we propose the following recommendations:

1. It is fruitful to investigate the suitable time, dose, and means of polyphenols to optimise its functions in a large number of animal models and in human subjects. Polyphenols are widely expressed in fruits and vegetables, and many epidemiological studies have shown that the consumption of these compounds via fruits and vegetables can reduce the incidence of a variety of chronic diseases. However, the actual results from the intervention experiments have differed from the expected results. Although the reasons for this discrepancy are not fully understood, they include potential differences in doses, interactions with the food matrix, and the differences in the bioavailability of polyphenols [79]. Therefore, an improved bioavailability would likely improve the beneficial effects of polyphenols in the host.
2. Polyphenols affect various mechanisms within different immunological responses, and thus, targeted immunotherapy requires an understanding of these mechanisms of action. Regarding immune protection, polyphenols can not only regulate the host immune system but also directly target the pathogen. To increase the efficacy of polyphenols, researchers must not only understand the immunological effects of different types of polyphenols but also determine the appropriate mechanism.
3. Different populations and age groups harbour different microbial populations, and the interactions between these microbes and immune cells are not negligible. Polyphenols play a vital role in the microbial community, as they have positive effects on the microbes. Simultaneously, these microbes promote the oxidation and degradation of polyphenols. Therefore, polyphenols may change the immune capacity of the host by altering the microbiota. In addition, polyphenols and conventional antimicrobial agents may exert synergistic effects on clinical multidrug-resistant microbes when administered in combination.
4. Notably, both the environment and polyphenol doses vary, and these variances are further affected by differences between *in vivo* and *in vitro* settings. Therefore, additional studies are needed to determine the immune responses to polyphenols in animal models, as well as the related health outcomes. Undoubtedly, the *in vivo* environment is more complex and malleable, compared to the *in vitro* environment. Accordingly, *in vitro* studies can only be used as references for *in vivo* experiments and cannot be used to determine the long-term effects of polyphenol supplementation on human health.

## References

[1] Williams A. R., Krych L., Ahmad H. F. (2017). A polyphenol-enriched diet and *Ascaris suum* infection modulate mucosal immune responses and gut microbiota composition in pigs. *PLoS One*.

[2] del Cornò M., Scazzocchio B., Masella R., Gessani S. (2016). Regulation of dendritic cell function by dietary polyphenols. *Critical Reviews in Food Science and Nutrition*.

[3] Szliszka E., Krol W. (2011). The role of dietary polyphenols in tumor necrosis factor-related apoptosis inducing ligand (TRAIL)-induced apoptosis for cancer chemoprevention. *European Journal of Cancer Prevention*.

[4] Ma Y., Kosińska-Cagnazzo A., Kerr W. L., Amarowicz R., Swanson R. B., Pegg R. B. (2014). Separation and characterization of soluble esterified and glycoside-bound phenolic compounds in dry-blanched peanut skins by liquid chromatography–electrospray ionization mass spectrometry. *Journal of Agricultural and Food Chemistry*.

[5] Pandey K. B., Rizvi S. I. (2009). Plant polyphenols as dietary antioxidants in human health and disease. *Oxidative Medicine and Cellular Longevity*.

[6] Mosele J. I., Macià A., Romero M. P., Motilva M. J., Rubió L. (2015). Application of *in vitro* gastrointestinal digestion and colonic fermentation models to pomegranate products (juice, pulp and peel extract) to study the stability and catabolism of phenolic compounds. *Journal of Functional Foods*.

[7] Correa-Betanzo J., Allen-Vercoe E., McDonald J., Schroeter K., Corredig M., Paliyath G. (2014). Stability and biological activity of wild blueberry (*Vaccinium angustifolium*) polyphenols during simulated *in vitro* gastrointestinal digestion. *Food Chemistry*.

[8] Magrone T., Jirillo E., Spagnoletta A. (2017). Immune profile of obese people and in vitro effects of red grape polyphenols on peripheral blood mononuclear cells. *Oxidative Medicine and Cellular Longevity*.

[9] Anisimov V., Popovich I., Zabezhinski M. (2016). Polyphenolic drug composition based on benzenepolycarboxylic acids (BP-C3) increases life span and inhibits spontaneous tumorigenesis in female SHR mice. *Aging*.

[10] Mohammad A. J., Mahdi N. R. (2017). Effect of grape seed polyphenol on immune gene expression and it’s role as antibacterial against *Salmonella typhimurium* infection in mice exposed to sodium nitrate. *International Journal of Advanced Research in Biological Sciences*.

[11] Cheynier V. (2005). Polyphenols in foods are more complex than often thought. *The American Journal of Clinical Nutrition*.

[12] Heleno S. A., Martins A., Queiroz M. J. R. P., Ferreira I. C. F. R. (2015). Bioactivity of phenolic acids: metabolites *versus* parent compounds: a review. *Food Chemistry*.

[13] Kim H. S., Quon M. J., Kim J. A. (2014). New insights into the mechanisms of polyphenols beyond antioxidant properties; lessons from the green tea polyphenol, epigallocatechin 3-gallate. *Redox Biology*.

[14] Romier B., Schneider Y. J., Larondelle Y., During A. (2009). Dietary polyphenols can modulate the intestinal inflammatory response. *Nutrition Reviews*.

[15] Szliszka E., Krol W. (2013). Polyphenols isolated from propolis augment TRAIL-induced apoptosis in cancer cells. *Evidence-based Complementary and Alternative Medicine*.

[16] Taofiq O., Calhelha R. C., Heleno S. (2015). The contribution of phenolic acids to the anti-inflammatory activity of mushrooms: screening in phenolic extracts, individual parent molecules and synthesized glucuronated and methylated derivatives. *Food Research International*.

[17] Goufo P., Pereira J., Moutinho-Pereira J. (2014). Rice (*Oryza sativa* L.) phenolic compounds under elevated carbon dioxide (CO_2_) concentration. *Environmental and Experimental Botany*.

[18] Goufo P., Trindade H. (2014). Rice antioxidants: phenolic acids, flavonoids, anthocyanins, proanthocyanidins, tocopherols, tocotrienols, *γ*-oryzanol, and phytic acid. *Food Science & Nutrition*.

[19] Park J. E., Cuong T. D., Hung T. M. (2011). Alkaloids from *Chelidonium majus* and their inhibitory effects on LPS-induced NO production in RAW264.7 cells. *Bioorganic & Medicinal Chemistry Letters*.

[20] Hussain T., Tan B. (2017). Modulatory mechanism of polyphenols and Nrf2 signaling pathway in LPS challenged pregnancy disorders. *Oxidative Medicine and Cellular Longevity*.

[21] Mapesa J. O., Waldschmitt N., Schmoeller I. (2011). Catechols in caffeic acid phenethyl ester are essential for inhibition of TNF-mediated IP-10 expression through NF-*κ*B-dependent but HO-1- and p38-independent mechanisms in mouse intestinal epithelial cells. *Molecular Nutrition & Food Research*.

[22] Wang L. C., Lin Y. L., Liang Y. C. (2009). The effect of caffeic acid phenethyl ester on the functions of human monocyte-derived dendritic cells. *BMC Immunology*.

[23] Liu G., Jiang Q., Chen S. (2017). Melatonin alters amino acid metabolism and inflammatory responses in colitis mice. *Amino Acids*.

[24] Švajger U., Jeras M. (2012). Anti-inflammatory effects of resveratrol and its potential use in therapy of immune-mediated diseases. *International Reviews of Immunology*.

[25] Chen S., Jiang H., Wu X., Fang J. (2016). Therapeutic effects of quercetin on inflammation, obesity, and type 2 diabetes. *Mediators of Inflammation*.

[26] Okuda T., Ito H. (2011). Tannins of constant structure in medicinal and food plants—hydrolyzable tannins and polyphenols related to tannins. *Molecules*.

[27] Magrone T., Kumazawa Y., Jirillo E. (2014). Polyphenol-mediated beneficial effects in healthy status and disease with special reference to immune-based mechanisms. *Polyphenols in Human Health and Disease*.

[28] Zhu D., Ma Y., Ding S., Jiang H., Fang J. (2018). Effects of melatonin on intestinal microbiota and oxidative stress in colitis mice. *BioMed Research International*.

[29] Tachibana H. (2011). Green tea polyphenol sensing. *Proceedings of the Japan Academy, Series B*.

[30] Sprangers S., Vries T. J.d., Everts V. (2016). Monocyte heterogeneity: consequences for monocyte-derived immune cells. *Journal of Immunology Research*.

[31] Yang C. S., Wang X. (2010). Green tea and cancer prevention. *Nutrition and Cancer*.

[32] Arce-Sillas A., Álvarez-Luquín D. D., Tamaya-Domínguez B. (2016). Regulatory T cells: molecular actions on effector cells in immune regulation. *Journal of Immunology Research*.

[33] Shim J. H., Choi H. S., Pugliese A. (2008). (−)-Epigallocatechin gallate regulates CD3-mediated T cell receptor signaling in leukemia through the inhibition of ZAP-70 kinase. *Journal of Biological Chemistry*.

[34] Ranjith-Kumar C. T., Lai Y., Sarisky R. T., Cheng Kao C. (2010). Green tea catechin, epigallocatechin gallate, suppresses signaling by the dsRNA innate immune receptor RIG-I. *PLoS One*.

[35] Wang H. K., Yeh C. H., Iwamoto T., Satsu H., Shimizu M., Totsuka M. (2012). Dietary flavonoid naringenin induces regulatory T cells via an aryl hydrocarbon receptor mediated pathway. *Journal of Agricultural and Food Chemistry*.

[36] Tomaszewski P., Kubiak-Tomaszewska G., Pachecka J. (2008). Cytochrome P450 polymorphism--molecular, metabolic, and pharmacogenetic aspects. II. Participation of CYP isoenzymes in the metabolism of endogenous substances and drugs. *Acta Poloniae Pharmaceutica*.

[37] Terao J., Murota K., Kawai Y. (2011). Conjugated quercetin glucuronides as bioactive metabolites and precursors of aglycone *in vivo*. *Food & Function*.

[38] Li L., Davie J. R. (2010). The role of Sp1 and Sp3 in normal and cancer cell biology. *Annals of Anatomy - Anatomischer Anzeiger*.

[39] Lee K., Lee Y. J., Ban J. O. (2012). The flavonoid resveratrol suppresses growth of human malignant pleural mesothelioma cells through direct inhibition of specificity protein 1. *International Journal of Molecular Medicine*.

[40] Gong S. Q., Sun W., Wang M., Fu Y. Y. (2011). Role of TLR4 and TCR or BCR against baicalin-induced responses in T and B cells. *International Immunopharmacology*.

[41] Cao H., Urban J. F., Anderson R. A. (2008). Cinnamon polyphenol extract affects immune responses by regulating anti- and proinflammatory and glucose transporter gene expression in mouse macrophages. *The Journal of Nutrition*.

[42] Liu G., Yu L., Fang J. (2017). Methionine restriction on oxidative stress and immune response in dss-induced colitis mice. *Oncotarget*.

[43] Loke W. M., Hodgson J. M., Proudfoot J. M., McKinley A. J., Puddey I. B., Croft K. D. (2008). Pure dietary flavonoids quercetin and (-)-epicatechin augment nitric oxide products and reduce endothelin-1 acutely in healthy men. *The American Journal of Clinical Nutrition*.

[44] Monagas M. (2009). Dihydroxylated phenolic acids derived from microbial metabolism reduce lipopolysaccharide-stimulated cytokine secretion by human peripheral blood mononuclear cells. *Br J Nutr*.

[45] Buckwalter M. R., Albert M. L. (2009). Orchestration of the immune response by dendritic cells. *Current Biology*.

[46] Svajger U., Obermajer N., Jeras M. (2010). Dendritic cells treated with resveratrol during differentiation from monocytes gain substantial tolerogenic properties upon activation. *Immunology*.

[47] Xu L., Xue J., Wu P. (2013). Antifungal activity of hypothemycin against *Peronophythora litchii* in vitro and in vivo. *Journal of Agricultural and Food Chemistry*.

[48] Lin W., Wang W., Wang D., Ling W. (2017). Quercetin protects against atherosclerosis by inhibiting dendritic cell activation. *Molecular Nutrition & Food Research*.

[49] Feuerer M., Hill J. A., Mathis D., Benoist C. (2009). Foxp3^+^ regulatory T cells: differentiation, specification, subphenotypes. *Nature Immunology*.

[50] Davis C. D., Ross S. A. (2007). Dietary components impact histone modifications and cancer risk. *Nutrition Reviews*.

[51] Cuevas A., Saavedra N., Salazar L., Abdalla D. (2013). Modulation of immune function by polyphenols: possible contribution of epigenetic factors. *Nutrients*.

[52] Attig L., Gabory A., Junien C. (2010). Early nutrition and epigenetic programming: chasing shadows. *Current Opinion in Clinical Nutrition and Metabolic Care*.

[53] Ueda Y., Wang M.-F., Irei A. V., Sarukura N., Sakai T., Hsu T.-F. (2011). Effect of dietary lipids on longevity and memory in the SAMP8 mice. *Journal of Nutritional Science and Vitaminology*.

[54] Park L. K., Friso S., Choi S. W. (2012). Nutritional influences on epigenetics and age-related disease. *Proceedings of the Nutrition Society*.

[55] Boyanapalli S. S., Tony Kong A. N. (2015). “Curcumin, the king of spices”: epigenetic regulatory mechanisms in the prevention of cancer, neurological, and inflammatory diseases. *Current Pharmacology Reports*.

[56] Borutinskaitė V., Virkšaitė A., Gudelytė G., Navakauskienė R. (2018). Green tea polyphenol EGCG causes anti-cancerous epigenetic modulations in acute promyelocytic leukemia cells. *Leukemia & Lymphoma*.

[57] Singh N., Dueñas-González A., Lyko F., Medina-Franco J.. L. (2009). Molecular modeling and molecular dynamics studies of hydralazine with human DNA methyltransferase1. *ChemMedChem*.

[58] Nandakumar V., Vaid M., Katiyar S. K. (2011). (-)-Epigallocatechin-3-gallate reactivates silenced tumor suppressor genes, *Cip1/p21* and p*16^INK4a^*, by reducing DNA methylation and increasing histones acetylation in human skin cancer cells. *Carcinogenesis*.

[59] Carthew R. W., Sontheimer E. J. (2009). Origins and mechanisms of miRNAs and siRNAs. *Cell*.

[60] Arola-Arnal A., Blade C. (2011). Proanthocyanidins modulate microRNA expression in human HepG2 cells. *PLoS One*.

[61] Milenkovic D., Deval C., Gouranton E. (2012). Modulation of miRNA expression by dietary polyphenols in apoE deficient mice: a new mechanism of the action of polyphenols. *PLoS One*.

[62] Xu X. R., Liu C. Q., Feng B. S., Liu Z. J. (2014). Dysregulation of mucosal immune response in pathogenesis of inflammatory bowel disease. *World Journal of Gastroenterology*.

[63] Xu G., Ren G., Xu X. (2010). Combination of curcumin and green tea catechins prevents dimethylhydrazine-induced colon carcinogenesis. *Food and Chemical Toxicology*.

[64] Banerjee N., Kim H., Talcott S. T., Turner N. D., Byrne D. H., Mertens-Talcott S. U. (2016). Plum polyphenols inhibit colorectal aberrant crypt foci formation in rats: potential role of the miR-143/protein kinase B/mammalian target of rapamycin axis. *Nutrition Research*.

[65] Okazaki Y., Han Y., Kayahara M., Watanabe T., Arishige H., Kato N. (2010). Consumption of curcumin elevates fecal immunoglobulin A, an index of intestinal immune function, in rats fed a high-fat diet. *Journal of Nutritional Science and Vitaminology*.

[66] Pérez-Berezo T., Franch A., Castellote C., Castell M., Pérez-Cano F. J. (2012). Mechanisms involved in down-regulation of intestinal IgA in rats by high cocoa intake. *The Journal of Nutritional Biochemistry*.

[67] Ramiro-Puig E., Pérez-Cano F. J., Ramos-Romero S. (2008). Intestinal immune system of young rats influenced by cocoa-enriched diet. *The Journal of Nutritional Biochemistry*.

[68] Zhang X., Zhivaki D., Lo-Man R. (2017). Unique aspects of the perinatal immune system. *Nature Reviews Immunology*.

[69] Lakhanpal D. P., Rai D. D. K. (2007). Quercetin: a versatile flavonoid. *Internet Journal of Medical Update - EJOURNAL*.

[70] Hirpara K. V., Aggarwal P., Mukherjee A. J., Joshi N., Burman A. C. (2009). Quercetin and its derivatives: synthesis, pharmacological uses with special emphasis on anti-tumor properties and prodrug with enhanced bio-availability. *Anti-Cancer Agents in Medicinal Chemistry*.

[71] Di Meo F., Lemaur V., Cornil J. (2013). Free radical scavenging by natural polyphenols: atom versus electron transfer. *The Journal of Physical Chemistry A*.

[72] Persia F. A., Mariani M. L., Fogal T. H., Penissi A. B. (2014). Hydroxytyrosol and oleuropein of olive oil inhibit mast cell degranulation induced by immune and non-immune pathways. *Phytomedicine*.

[73] Choi Y. H., Yan G. H. (2009). Silibinin attenuates mast cell-mediated anaphylaxis-like reactions. *Biological and Pharmaceutical Bulletin*.

[74] Sato Y., Akiyama H., Matsuoka H. (2010). Dietary carotenoids inhibit oral sensitization and the development of food allergy. *Journal of Agricultural and Food Chemistry*.

[75] Green D. R., Ferguson T., Zitvogel L., Kroemer G. (2009). Immunogenic and tolerogenic cell death. *Nature Reviews Immunology*.

[76] Krysko D. V., Garg A. D., Kaczmarek A., Krysko O., Agostinis P., Vandenabeele P. (2012). Immunogenic cell death and DAMPs in cancer therapy. *Nature Reviews Cancer*.

[77] Gomez-Cadena A., Urueña C., Prieto K. (2016). Immune-system-dependent anti-tumor activity of a plant-derived polyphenol rich fraction in a melanoma mouse model. *Cell Death & Disease*.

[78] Yi L., Zongyuan Y., Cheng G., Lingyun Z., GuiLian Y., Wei G. (2014). Quercetin enhances apoptotic effect of tumor necrosis factor-related apoptosis-inducing ligand (TRAIL) in ovarian cancer cells through reactive oxygen species (ROS) mediated CCAAT enhancer-binding protein homologous protein (CHOP)-death receptor 5 pathway. *Cancer Science*.

[79] Bohn T. (2014). Dietary factors affecting polyphenol bioavailability. *Nutrition Reviews*.

